# The potential role of temporal dynamics in approach biases: delay-dependence of a general approach bias in an alcohol approach-avoidance task

**DOI:** 10.3389/fpsyg.2014.01398

**Published:** 2014-12-01

**Authors:** Thomas E. Gladwin, Sören E. Mohr, Reinout W. Wiers

**Affiliations:** Addiction Development and Psychopathology Lab, Department of Developmental Psychology, University of AmsterdamAmsterdam, Netherlands

**Keywords:** automatic, approach bias, src, delay

## Abstract

Attractive cues have been shown to evoke automatic approach biases in tasks such as the Automatic Approach Task or Stimulus Response Compatibility task. An important but as yet not studied question is the role of temporal dynamics in such tasks: the impact of automatic processes may depend on the interval between cue and response. The current proof of principle study tested this hypothesized time-dependence of the approach bias. Secondary goals included the exploration of effects of alcohol cues and virtual hand stimuli. 22 participants performed an SRC task in which the delay between the presentation of the cue and the possibility to select the response was manipulated. Results revealed an approach bias that decayed over longer delays. Thus, the approach bias was indeed dependent on processes that are transiently evoked by cues. The results did not show significant effects of alcohol cues or a virtual hand. Temporal dynamics may be an essential feature of approach biases.

## INTRODUCTION

Relatively attractive, appetitive cues, such as drug-related stimuli, have been shown to evoke automatic approach tendencies in versions of tasks such as the Approach Avoidance Task (AAT) or manikin Stimulus Response Compatibility (SRC) task (Saraiva et al., 2013; Phaf et al., 2014). In the AAT, participants typically use a joystick to pull a stimulus toward them or push a stimulus away from them. This allows an approach/avoidance bias for one stimulus category relative to another to be measures; arachnophobic subjects are for example relatively slow to pull images of spiders toward them (Rinck and Becker, 2007), and drug-using subjects are relatively fast to pull drug-related stimuli (Wiers et al., 2009; Cousijn et al., 2011). In the SRC, the stimulus to be approached or avoided is presented in the center of the screen, and a movable Response-stimulus – typically a manikin stick figure – is moved toward or away from the stimulus. Using the SRC, effects for negative versus positive words and spiders have been demonstrated (Krieglmeyer and Deutsch, 2010), and heavier drinkers have been shown to be relatively fast to approach alcohol stimuli (Field et al., 2008, 2011). Approach effects have also been found on attention to alcohol cues, indicating that such cues are salient and attractive to drinkers: for instance, heavy social drinkers show an attentional bias toward alcohol cues (Townshend and Duka, 2001), although such effects are complex (see below and, e.g., Loeber et al., 2009).

A potentially important but as yet understudied aspect of automatic approach biases is their temporal dynamics. Recent theoretical perspectives suggest that temporal dynamics may play a fundamental role in the contrast between reflective and automatic processing (Cunningham et al., 2007; Gladwin et al., 2011; Gladwin and Figner, 2014). Indeed, highly time-dependent effects have been found for attentional biases for alcohol, in which an engagement – disengagement temporal sequence following alcohol cues has been found (Noël et al., 2006; Townshend and Duka, 2007; Vollstädt-Klein et al., 2009). In these tasks, trials begin with a task-irrelevant cue stimulus shown onscreen: a picture of an alcoholic beverage, or a non-alcoholic beverage. This is followed by a variable delay period, after which a probe stimulus, to which subjects must execute a speeded choice response, appears. The probe appears either at the same location of the first stimulus or at a different location. If the delay period is brief (around 150 ms), alcohol-dependent subjects are faster to respond to probes if they appear at the location of alcoholic cue stimuli, relative to non-alcoholic cue-stimuli. After longer delay periods (around 600 ms), this effect is reversed: subjects are then relatively slow to respond to probes at the location of alcoholic cues. This is in line with attention initially being drawn to alcohol cues, but subsequently moved away. One interpretation of this effect is that subjects cannot completely avoid an initial attentional bias toward the salient alcohol cues, but have learned to shift attention away from them as soon afterward as possible, because such cues are able to capture attention and cause distraction and subsequent undesired behavior. Indirect evidence for this view has been found using fMRI, in which subjects with more problems with hazardous drinking showed decreased activation, when confronted with distracting alcohol stimuli, in a brain region associated with attentional control (Gladwin et al., 2013). In the context of working memory, distracting effects of alcohol distractors on subsequent task performance also have been found to depend on the interval between distractors and task stimuli (Gladwin and Wiers, 2011): alcohol stimuli in a secondary task cause a relatively prolonged distracting effect on performance. However, to our knowledge temporal effects have not been studied in the context of the alcohol approach bias in motor responses. When an alcohol cue is perceived, an approach response bias is expected (see e.g., Wiers et al., 2009; Field et al., 2011), but does this bias persist indefinitely, dissipate, or reverse as found for attentional biases?

The primary goal of the current study was therefore to provide a first step in determining the time-dependence of approach biases in an SRC. To this aim, we developed an Alcohol-Approach Task in which responses could not be selected or executed until a given delay following the cue. A second, more exploratory goal was to compare effects when using of a “virtual hand” as the movable response-stimulus, instead of the usual abstract manikin. From the perspective of embodied cognition (Garbarini and Adenzato, 2004), approach biases for appetitive stimuli may be intimately related to physical, bodily actions, such as grasping or moving the hand away from stimuli. Further, previous research has shown that subjects can experience a sense of vicarious agency of others’ hands when their movements were associated with congruent instructions (Wegner et al., 2004). We therefore hypothesized that subjects would exhibit stronger biases when moving a representation of a hand rather than a more abstract stimulus. This would more closely represent the actual act of grasping an alcoholic beverage and thereby potentially lead to enhanced effects, if in fact such effects depend on this closeness of representation.

## MATERIALS AND METHODS

### PARTICIPANTS AND PROCEDURE

Participants were 24 college students (four male, mean age 22, SD = 3) who participated for money (7 €) or course credit. One subject was lost due to technical problems, and one subject did not correctly perform the task, leaving 22 subjects for analysis. One subject did not complete the full session; removing or including this subject did not substantially affect results. Participants signed an informed consent form and the study had the necessary IRB approval from the University of Amsterdam Ethical Committee. Subjects received verbal and written instructions and were seated in front of a PC to perform tasks: a Preference task, the SRC task, and another task not reported here. After performing the tasks, subjects filled in the AUDIT questionnaire on risky drinking (Saunders et al., 1993). The mean AUDIT score was 6.5 (SD = 3.3), which is in the high range of low-risk drinking (Saunders et al., 1993).

### TASKS

#### Preference task

The first task was used to individualize stimuli. Subjects were presented with pairs of stimuli shown next to each other. Stimuli were drawn from a set of 24 color pictures of beverages (12 alcoholic, 12 non-alcoholic). Subjects chose which of the beverages they most preferred. After the first selection, subjects were presented with one novel picture, which was consecutively paired with the most to least preferred stimuli so far, until it was either selected or found to be less preferred than the previously least preferred picture, thus sorting the stimuli. The four most preferred alcohol stimuli and the four most preferred soft drink stimuli were selected as stimuli in subsequent tasks.

#### Manikin SRC task

The Manikin task consisted of eight blocks of twenty trials. Prior to each block, subjects received instructions on how to respond in the upcoming trials. Instructions were either to approach alcohol and avoid soft drink stimuli, or avoid alcohol and approach soft-drink stimuli. Trials began with the presentation of a centered beverage stimulus (visual angle around 14°). After a delay of 0, 300, 600, or 900 ms, a manikin appeared to the left or right of the beverage stimulus. Delays were selected at random per trial, which resulted in a mean proportion of trials with a given delay of 0.25 as expected, with a SD of 0.026 and a minimum proportion of 0.20 trials and a maximum proportion of 0.32 trials over all subjects. Subjects were to move the manikin figure toward or away from the beverage stimulus, depending on the current block instructions. If the manikin appeared to the right of the beverage stimulus, a single press of the J or K key (index and middle finger) was used to move the manikin toward and away from the stimulus, respectively. If the manikin appeared to the left, the F and D keys were used to move the figure. Thus the hand was used that matched the location of the manikin, and the finger of the hand was used that matched the direction of the movement of the movement. After a button press, the manikin was animated to move in the indicated direction, either to the side or the center of the screen, taking one second. If an incorrect response was given the word “Incorrect” was displayed in red for 2000 ms. Trials ended with a 200–300 ms inter-trial interval during which a fixation cross was presented.

#### Hand task

The Hand task was identical to the Manikin task, except that a photographed image of a hand, in an open grasping position, replaced the manikin figure. The hand was shown roughly at the angle at which the subject’s own hand would be viewed if it were placed on a table. A left or right hand was presented depending whether the hand appeared to the left or to the right of the beverage stimulus.

### STATISTICAL ANALYSIS

The first four trials of the task, the first trial per block, and trials with reaction times below 150 ms or above 1500 ms were excluded from analysis. Reaction time (RT) and accuracy of responses to the appearance of the manikin or hand were analyzed using repeated measures MANOVA. One MANOVA tested the differences between RT scores over different conditions for the different within-subject conditions; another MANOVA tested the accuracy scores. The within-subject factors for each MANOVA were Response-Stimulus (Hand versus Manikin), Alcohol (alcoholic versus soft drink cue), Approach (required approach versus avoid response) and Delay (cue – response-stimulus interval: 0, 300, 600, or 900 ms).

## RESULTS

Reaction times and accuracies are presented in **Table 1**. The following effects on RT were found. Approach responses were significantly faster than avoid responses [*F*(1,21) = 13.14, *p* = 0.002, $\eta_{p}^{2}$ = 0.39]. Increasing delays were associated with faster responses [*F*(3,19) = 282.91, *p* < 0.0005, $\eta_{p}^{2}$ = 0.98]. *Post hoc* analyses comparing all six pairs of CSIs were performed using two-sided *t* tests. The following pairs had significant difference at a criterion of 0.05/6, i.e., using Bonferroni correction for the number of pairs to be tested (choosing 2 CSIs from the set of 4 CSIs; the same pairs were significant at a 0.05 criterion): all other CSIs versus 0 ms and both 900 ms and 600 ms versus 300 ms.

**Table 1:** Behavioral results.

	Manikin				Hand			
	Soft		Alcohol		Soft		Alcohol	
	Avoid	Approach	Avoid	Approach	Avoid	Approach	Avoid	Approach
Reaction time (RT)	535.16 (74)	511.41 (80)	514.3 (70)	511.7 (74)	525.58 (87)	496.9 (73)	521.97 (69)	502.27 (85)
Accuracy	0.93 (0.046)	0.94 (0.044)	0.94 (0.043)	0.93 (0.042)	0.91 (0.059)	0.94 (0.043)	0.93 (0.044)	0.94 (0.041)

*Reaction time and accuracy data. RT, mean (SD) reaction times in ms. Accuracy, proportion of accurate responses. Manikin and Hand refer to the task versions, in which subjects moved a manikin or a virtual hand, respectively. Soft and Alcohol refer to the beverage type to approached or avoided*.

**FIGURE 1:**
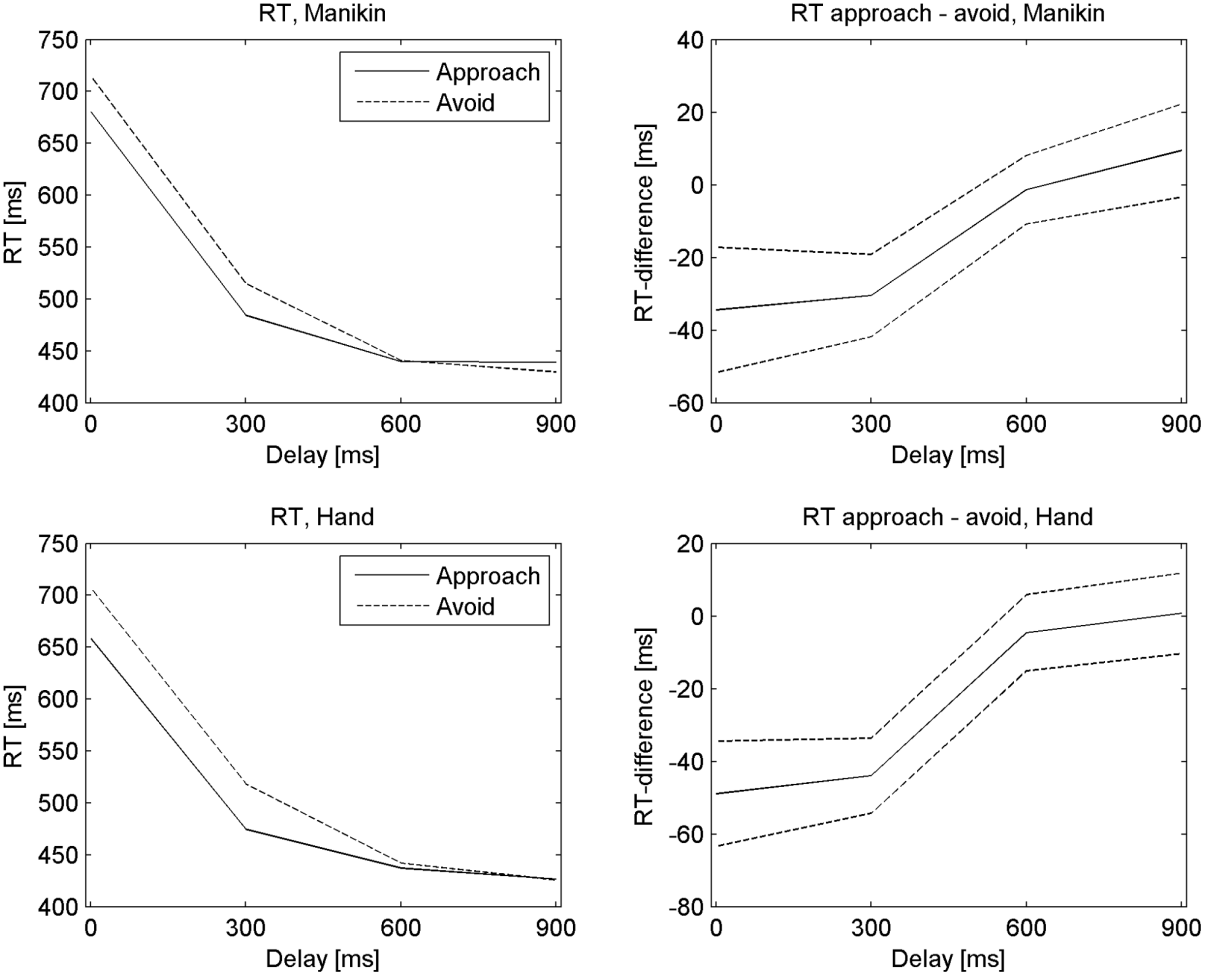
**The decay of the approach bias over increasing cue-stimulus interval.** Reaction times (left column) and approach – avoid differences (right column) for the Hand and Manikin versions of the task (top and bottom row, respectively). SEs are shown as dotted lines for difference scores in the right column. Note that the specific motor response was only known at the time of Stimulus presentation, yet the approach bias depended strongly on the time since the presentation of the preceding Cue.

Essentially, the effects of Approach and Delay interacted [*F*(3,19) = 9.90, *p* < 0.0005, $\eta_{p}^{2}$ = 0.61], due to decreasing approach biases for higher delays (**Figure 1**). *Post hoc* analyses of the Approach by Delay interaction were performed by testing differences in approach bias (i.e., the approach minus avoid difference score) between pairs of delays. Significant decreases over increased delay were found between 600 ms versus 300 ms, 900 versus 0 ms, and 900 versus 300 ms (all *p* < 0.05/6; additionally, for 300 ms versus 0 ms, *p* = 0.014). Thus, the set of the two shorter delays differed from the set of the two longer delays, but no significant difference was found between 0 and 300 ms delay, or between 600 and 900 ms delay.

On accuracy, only an effect of Delay was found [*F*(3,19) = 4.71, *p* = 0.013, $\eta_{p}^{2}$ = 0.43]. *Post hoc* two-sided *t* tests were performed, correcting for the six pairwise comparisons as for RT. Accuracy was higher for 600 ms and 900 ms versus 0 ms CSI; additionally, for 300 versus 0 ms, *p* = 0.03.

## DISCUSSION

As hypothesized, approach biases decreased with delays, in line with the idea that the balance between reflective, task-related processing and automatic biases may involve time-dependent processes. Note that, essentially, if it were the case that the approach bias involves only a process involving execution of the movement itself, it should not depend on the time since cue presentation. In contrast, it appears that cues transiently evoke approach tendencies, independent of response execution. Evidence for a role of similar temporal dynamics in attentional tasks have been found previously (Noël et al., 2006; Townshend and Duka, 2007; Vollstädt-Klein et al., 2009; Gladwin and Wiers, 2011) and they may play a fundamental role in automatic processes related to, e.g., drug-related approach biases (Gladwin et al., 2011). If so, understanding these time-dependent effects may have clinical implications: if approach tendencies decay relatively quickly, training subjects to even slightly delay responses to drug stimuli may be effective in reducing approach behavior, possibly playing a role in the efficacy of interventions such as cognitive bias modifications using approach – avoidance retraining (Wiers et al., 2011).

We note a number of limitations of the current study. The time-dependent effects in the current study were not specific to alcohol stimuli or risky drinking. The approach bias was found for both beverage types, which may reflect general appetitive attributes of both soft drinks and alcoholic drinks for this population. Indeed, in previous research a gene-dependent approach bias was also found for both alcoholic and non-alcoholic appetitive stimuli (Wiers et al., 2009). Therefore we cannot attribute the current time-dependent approach bias to alcohol-specific processes. More delay intervals, allowing more fine-grained analyses, and the inclusion of heavier drinkers may yet reveal speed-of-decay effects that are related to alcohol. Rating scales could be added to the stimulus selections procedure in order to capture more information about the relative subjective values of the stimulus categories. Although beyond the scope of the current study, it would also be interesting to include cues evoking avoidance biases in future research, such as phobia-related or unpleasant stimuli. With such stimuli, an initial avoidance bias would be expected that decays with time, or possibly reverses to approach as control is exerted.

In the current study, delays were selected randomly per trial. Although this does not seem likely to have influenced the findings, future research should more precisely control for the number of trials per delay period.

No effects of the response-stimulus, a manikin or a hand, were found. Possibly, both stimuli led to a similar coding of responses as approach versus avoidance. Future research could yet explore this further by using actual grasping motions rather than key presses, but the current results do not seem to provide evidence for likely strong differences. If this null result proves robust, it would suggest that the neural representations of approach/avoidance that lead to biases are not closely tied to specific motor representations.

In conclusion, approach biases appear to reflect time-dependent processes that decay after cue presentation. Thus, the manipulation of the timing of responses relative to cue presentation may be of potential importance for the study of approach-avoidance biases. We did not find evidence that using stimuli that closely reflect actual body movements may be better able to evoke motivational behaviors than more abstract stimuli, although it is as yet uncertain whether different circumstances such stimuli could yet lead to significantly different effects. Studies focused on more fine-grained analyses of effects of delay would appear to be a potentially fruitful line of research, and could open novel methods such as the analysis of the speed of decay of biases.

## AUTHOR CONTRIBUTIONS

Thomas E. Gladwin developed the aims of the experiment, designed and programmed the task and performed the statistical analyses. Thomas E. Gladwin, Sören E. Mohr and Reinout W. Wiers conducted literature searches and contributed to writing the manuscript. Sören E. Mohr recruited and measured subjects.

## Conflict of Interest Statement

The authors declare that the research was conducted in the absence of any commercial or financial relationships that could be construed as a potential conflict of interest.
